# Categorizing US State Drinking Practices and Consumption Trends

**DOI:** 10.3390/ijerph7010269

**Published:** 2010-01-20

**Authors:** William C. Kerr

**Affiliations:** Alcohol Research Group, Public Health Institute, 6475 Christie Avenue, Suite 400, Emeryville, CA 94608, USA; E-Mail: wkerr@arg.org; Tel.: +1 510 597-3440; Fax: +1 510 985-6459

**Keywords:** alcohol, regional, United States, drinking pattern

## Abstract

US state alcohol consumption patterns and trends are examined in order to identify groups of states with similar drinking habits or cultures. Rates of heavy drinking and current abstention and *per capita* apparent consumption levels are used to categorize states. Six state groupings were identified: North Central and New England with the highest consumption and heavy drinking levels; Middle Atlantic, Pacific and South Coast with moderate drinking levels; and Dry South with the lowest drinking levels. Analyses of relationships between beer and spirits series for states within groups as compared to those in different groups failed to clearly indicate group cohesiveness.

## 1. Background

Each state of the United States has unique characteristics relevant to the determination of a drinking culture and corresponding alcohol consumption patterns and alcohol-related problems. Factors that may underlie or influence drinking culture include a state’s demographic make-up in terms of age, gender and race/ethnicity groups, the distribution of socio-economic characteristics such as educational attainment, income and wealth, a state’s mix of religions with differing perspectives on alcohol use, degree of urbanicity, state alcohol policies and regulatory regimes, the structure of the alcoholic beverage market in terms of wholesale and retail distribution networks, historical and cultural practices related to drinking (or not drinking) various alcoholic beverages, the size and importance of alcoholic beverage production industries both historically and currently, exposure to advertising for alcohol products and potentially other factors. Yet, clearly some of these aspects of drinking culture are shared across states, particularly those in geographic proximity to one another, and other aspects may be shared across states with similar populations throughout the country. Therefore, one would expect that drinking cultures and markets would not be defined by state borders in most cases. Media markets too may help create cultural sub-groups that cover multiple states or even market-related areas potentially spread across many parts of the country. Relative proportions of the various demographic and market-demarcated sub-groups, along with geographic proximity, should define the degree of shared drinking cultures between states. Thus, states may share similarities in terms of certain beverage types or drinking patterns but may be divergent on other beverages or trends. It is also relevant regarding drinking culture, to consider commonalities reflected in trends over time.

Past research has generally focused on characterizing Wet and Dry regions of the US. In these studies and the current analysis “wetness” is determined by consumption patterns with Wet areas having a relatively high *per capita* consumption and percentage of heavy drinkers and a relatively low abstention rate. Alternative definitions of “wetness” in terms of alcohol policy and alcohol availability could also be applied and future studies should address how these are related to the drinking outcome-based definition of wetness. Early work utilizing data from the first National Alcohol Survey (NAS) conducted in 1964 using nine Census-defined regions found the Northeast, North Central and Pacific regions to be Wet and the South, Mountain and West North Central states to be Dry [1]. Further analyses looking at the same nine census-defined regions in NAS surveys from 1979 and 1984 showed the drier regions had become wetter since the 1960s but that Dry/Wet differences in both drinking patterns and attitudes remained [1,2]. In these analyses the West North Central region was reclassified from Dry to Wet and the West South Central and Mountain regions remained Dry in terms of abstention rates but looked more like Wet regions in terms of heavy drinking and *per capita* consumption.

Survey measures of consumption volume have been found to be highly correlated with *per capita* apparent consumption [3] and survey estimates of drinking by college students have been found to be linked to drinking patterns in the general population [4]. Some have suggested that the Southern states appear to be dry by having higher abstention rates but actually have heavier drinking among those who drink; however, an analysis of survey data across states did not find differences between the South and other states in terms of heavy drinking among drinkers [5]. This may be because the marginal drinkers who choose to abstain in a Dry environment would have been very light drinkers in a Wet environment, making per drinker average consumption a poor measure for comparison due to the typically skewed consumption distribution [6]. Religion has been found to be an important predictor of current drinking and heavy drinking across US states with high religious adherence for Catholics being associated with higher rates of drinking and heavy drinking, and higher adherence among Evangelical Protestants associated with lower rates [7].

Survey-based models of the determinants and consequences of alcohol use typically utilize regional control variables to capture unmeasured aspects of drinking culture [8]. Most US regional control variables in multivariate models utilize either the nine or four census regions. Such analyses are motivated by the notion that some variance is explained by characteristics associated with simple geography. Having a set of such geographic variables based on empirical measures characterizing states’ drinking cultures should improve their usefulness. Analyses retaining indicators for all 50 states are often not informative or feasible. Aggregate-level cross-section time-series models must pool states’ series in many cases, for example when estimating the effects of government policies on alcohol use or the effects of alcohol consumption on mortality rates or in modeling other outcomes. Knowing which states are similar to each other in terms of drinking culture, broadly defined, could improve the selection of control states in models of interrupted time series aimed at detecting results of a policy change, for instance. In either survey or aggregate analyses it would be preferable to utilize groups of states that have been shown to have similar and linked drinking markets and/or cultures than to employ arbitrarily composed contiguous geographic regions. An improved grouping scheme should result in better precision of regional indicator variables for survey analyses and ideally yield more homogeneous pooled state groupings for time-series analyses.

No studies have specifically tried to group states empirically based on drinking pattern and alcohol sales data. Much more state-specific data is now available to fine tune these characterizations and to begin to empirically define state groupings in terms of shared drinking culture. Measures characterizing drinking culture in this study focus on the percentage of the population drinking five or more (5+) drinks in a day in the past month, drinking at all in the past month and *per capita* apparent alcohol consumption. This paper aims to categorize the US states into regional groups based on the wetness of recent drinking patterns and to evaluate whether these groupings represent linked drinking cultures over time through pair-wise analyses of trends in *per capita* apparent consumption of beer and spirits within and outside of these regional groupings.

## 2. Methods

### 2.1. Data Sources

Survey estimates for each state are taken from synthetic state-level estimates for the 2005–2006 combined samples from the National Survey on Drug Use and Health (NSDUH) of past month 5+ drinks in a day and past month drinking among those aged 12 and older and other age sub-groups. These published estimates were used because to prevent identification, the public use files for this survey do not include geographic identifiers [9]. The NSDUH survey is a face-to-face multi-stage clustered probability sample of the US population with special attention to drug use related issues. Alternative estimates of 5+ for men and 4+ for women drinks in a day in the past month from the Behavioral Risk Factor Surveillance System (BRFSS) in 2007 are shown in Table 1 below for comparison. The BRFSS survey utilizes state representative sampling; each state oversees the conduct of an identical, large telephone survey on health risk behaviors [10].

*Per capita* (aged 15 and older) apparent alcohol consumption data for 1950 to 2002 and for 2005 are estimates from the Alcohol Research Group (ARG) state alcohol *per capita* ethanol database. Tax and sales based estimates of per capita apparent consumption of beer, wine, spirits and total ethanol for 1950 to 2005 are calculated using %ABV conversions developed by ARG and population estimates are from the US Census Bureau [11–13].

### 2.2. Methodology

State groupings were created based on geographic proximity and primarily on the estimate, from the NSDUH 2005–2006 combined sample, of the percentage of those 12 and older drinking 5+ drinks in a day on any day in the past month, as well as from the 2005 *per capita* apparent consumption of alcohol. Also considered were the past month 5+/4+ binge measure taken form the 2007 BRFSS survey and the past year abstention rate in the 2005–2006 NSDUH surveys. The NSDUH estimates are seen as more reliable than the BRFSS estimates due to higher response rates and especially higher reported volumes of drinking, implying better coverage of alcohol sales [14]. *Per capita* apparent consumption figures are generally seen as the most reliable estimates of overall drinking, not being subject to non-response and self-report biases. However, an exception to this rule is states with large cross-border and/or tourist sales relative to their populations, particularly, Nevada, Alaska, Hawaii, District of Columbia, New Hampshire and Delaware. Correlations between reporting 5+ drinks in a day in the last month and monthly drinkers in the 2005–6 NSDUH, 2005 *per capita* apparent consumption and 5+/4+ drinks in a day monthly based on the 2007 BRFSS were also calculated to determine the degree to which these measures indicated similar rankings of states. The percentage of past month drinkers reporting having 5+ drinks in any day in the past month in the 2005–6 NSDUH was also calculated to illustrate the prevalence of heavy drinking among drinkers. Based primarily on inspecting the proportion of heavy drinkers and *per capita* apparent consumption of alcohol, but also considering the other measures, states were generally characterized as Wet, Moderate or Dry. States within these categories were then grouped according to geographic proximity with some exceptions made in order keep the number of groups relatively small and to include nearly all states in some group. For example, Utah was grouped with the other Dry states that form a geographically contiguous group in the South and the Moderate states in the South were grouped together although they do not all share borders.

Following the grouping, based on cross-sectional results, data on trends in *per capita* apparent consumption by beverage type were examined to give an indication of whether alcohol consumption trends within the state groupings appear to move together and to have similar levels and general trends. To establish whether relationships between states within a resultant regional grouping where stronger and more common than those with states outside of the groupings, Granger Causality tests [15] were estimated using Stata [16] between each pair of states for beer and spirits series. Wine was not analyzed because summarizing test results was very labor intensive and wine is a relatively less important beverage in the US. Granger Causality tests use lagged values of one series (e.g., *per capita* beer consumption in Ohio) to predict the current value of another series (e.g., *per capita* beer consumption in Iowa). These tests incorporated two lagged values and a 0.05 significance level was used for rejecting the null hypothesis of no relationship. The proportion of significant relationships (in either or both directions) between states within a group was then compared to the proportion with states outside of the group to determine the relative degree to which beverage series trends were more closely related within regional groups.

## 3. Results

**Figure 1.**
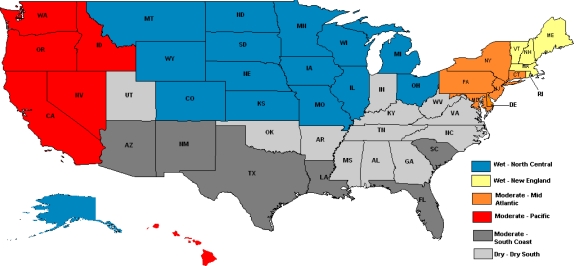
Map of US Regional Drinking Groups. North Central, blue; New England, yellow; Middle Atlantic, orange; Pacific, red; South Coast, dark grey; Dry South, light gray. Groups are also identified as Wet, Moderate or Dry based on the % reporting 5+ drinking day in the past month and *per capita* apparent consumption of alcohol.

Six state groups were identified by consideration of the variables mentioned in Methods, as shown in Figure 1. With group titles that approximate the bulk of the included states, these groups are:

Wet
  *North Central-* Alaska, Colorado, Illinois, Iowa, Kansas, Michigan, Minnesota, Montana, Missouri, Ohio, Nebraska, North Dakota, South Dakota, Wisconsin, Wyoming
  *New England-* Maine, Massachusetts, New Hampshire, Rhode Island, Vermont
Moderate
  *Middle Atlantic*- Connecticut, Delaware, Maryland, New Jersey, New York, Pennsylvania
  *Pacific*- California, Hawaii, Idaho, Nevada, Oregon, Washington
  *South Coast*- Arizona, Florida, Louisiana, New Mexico, South Carolina, Texas
Dry
  *Dry South***-** Alabama, Arkansas, Georgia, Indiana, Kentucky, Mississippi, North Carolina, Oklahoma, Tennessee, Utah, Virginia, West Virginia

The District of Columbia was not grouped due to unique characteristics but if a complete grouping is necessary for analytic purposes it would be included in the Middle Atlantic group. The District of Columbia is situated between Maryland in the Middle Atlantic and Virginia in the Dry South but has much higher alcohol consumption than both and differs in many ways, reflecting its unique features. Other states were also difficult to clearly classify. Nevada has increased in the number residents relative to visitors recently and now fits better into the Pacific region while Utah’s drinking pattern fits the Dry South although its drinking culture may not be closely linked to the other member states due to geographic and cultural separation. New Hampshire is another special case where out-of-state buyers inflate *per capita* apparent consumption estimates but residents’ drinking in surveys clearly fits with the New England group. Alaska also has a relatively high tourist/visitor impact and being geographically separated from the contiguous mainland states it is difficult to place its drinking culture in this environment. Figure 1 provides a visual presentation of these groups. Following this, Table 1 lists the percentage of the population drinking 5+ drinks in a day in the 2005–6 NSDUH for the age 12 and older group (used in the state grouping decision) and for sub-groups by age of 18 to 25 and 26 and older. Also included are the past month percentage of drinkers and the percentage reporting 5+ days among past month drinkers in the 2005–6 NSDUH, *per capita* apparent consumption for 2005 and the percentage of the population 18 and older reporting past month 5+/4+ days in the 2007 BRFSS survey. The states are listed in descending order by the past month 5+ percentages for those 12 and older in the 2005–6 NSDUH. Considering the regional groupings using the group name variable in terms of the ordering by 5+ percentages shows the groups to make sense in terms of this ordering. The US average is included for reference and all but one (Alaska) of the North Central states and all but one (Maine) of the New England states are seen to be above this average. These are the two groups of states represented as Wet. The Dry South states, seen as the Dry group, are all found below the US average. The three middle groups, Pacific, Middle Atlantic and South Coast are more distributed both above and below and are generally categorized as Moderate in terms of wetness.

Comparing the NSDUH estimates to those from the BRFSS and to *per capita* apparent consumption illustrates a general but far from complete agreement (see Table 1). It is clear that the BRFSS estimates are much lower than the NSDUH despite using a four drink threshold for heavy drinking among women and having an age 18 and older, rather than 12 and older, sampling frame. At face, both of these differences would be expected to lead to higher numbers in the BRFSS rather than lower. Clearly, some combination of differences in the sampling strategy, response rate, mode of interview, incentive payment and survey content led to less under-reporting in the NSDUH and may also imply more stable estimates across states due to lower measurement error. Several of these potential sources of bias were examined in a comparison of the 1999 and 2001 versions of each of these surveys but none of the factors examined appeared to explain these differences [17]. The *per capita* apparent consumption estimates are assessments of a state’s average consumption volume rather than the number of heavy drinkers and present an alternative metric for state comparisons. Because this was an important variable considered in group assignment decisions, making allowance for the states where this measure is biased by out-of-state drinkers, *per capita* consumption generally fits the state groupings. However, some states like Ohio, Kansas and Iowa in the North Central group appear lower on this measure indicating perhaps less moderate drinking along with a culture of heavy drinking. Similarly, some of the states ranking lower in 5+ percentage appear higher in *per capita* consumption, particularly those not in the Dry South group such as South Carolina, Oregon and New Mexico.

Correlations between the survey measures considered in the state group classifications were high with a 0.73 correlation between the 2005–6 NSDUH 5+ measure and the 2007 BRFSS 5+/4+ measure and a 0.82 correlation between the monthly 5+ and monthly drinking measures in the 2005–6 NSDUH. A lower 0.44 correlation between the NSDUH 5+ measure and *per capita* apparent consumption in 2005 indicates some disparity between these two key measures. However, when states with cross border and tourism issues are removed the correlation increases to 0.62, indicating somewhat better agreement between these measures. Table 1 also illustrates that while there is a general correspondence between the 18–25 age group and the older group in terms of 5+ drinkers, some states stand out particularly in the 18–25 group. The New England states are especially high in this group and are known for having relatively large numbers of college students and the associated heavy episodic college drinking cultures. Some Middle Atlantic states, such as New Jersey, Delaware and Pennsylvania, similarly have relatively high percentages of past month 5+ drinkers in the 18–25 group. The percentage of past month drinkers who report having had 5+ drinks in a day gives an indication of the distribution of heavy and moderate drinking among drinkers for each state. These percentages range from a low of 38% in Maryland to a high of 54% in Utah, both states with relatively low levels of 5+ drinkers. While there is no consistent pattern for this variable, higher percentages appear more common among states with the highest and lowest percentages of 5+ drinkers in their populations while lower percentages more commonly occur in the Moderate states.

**Figure 2.**
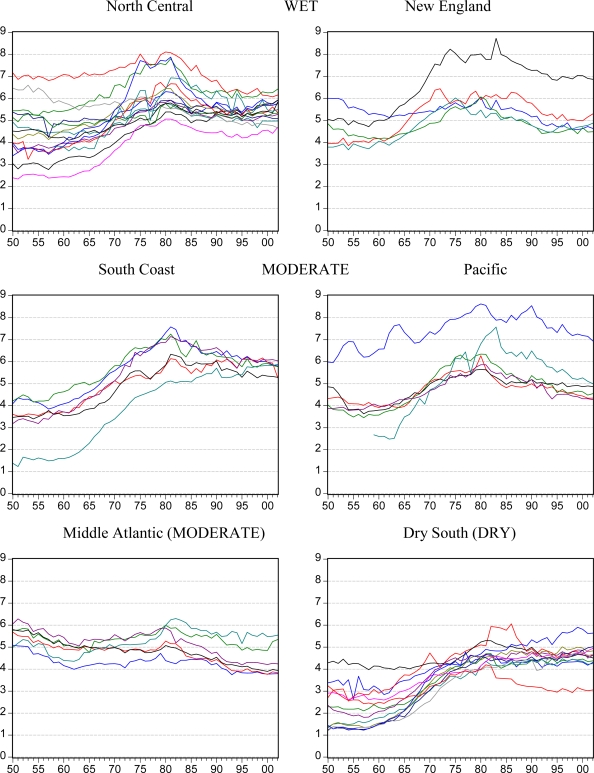
Apparent consumption of beer in liters of ethanol *per capita* aged 15+ by state in groups over the years from 1950 to 2002.

**Figure 3.**
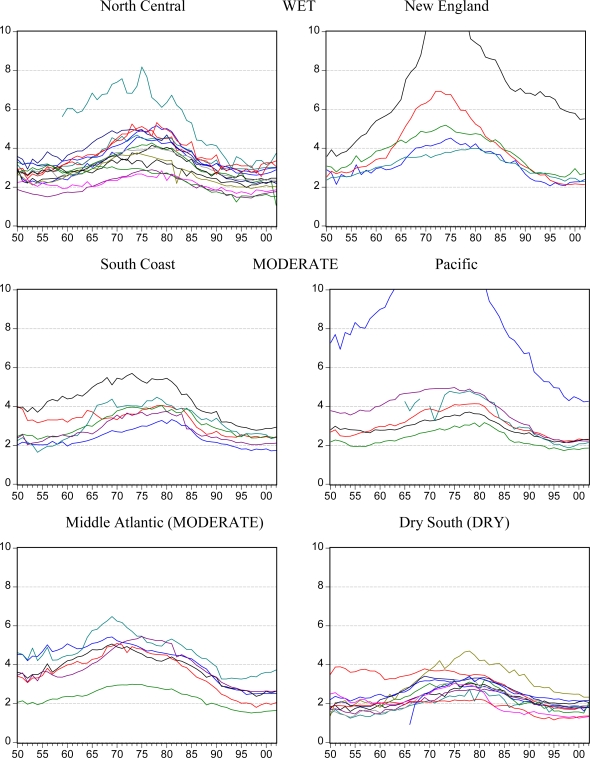
Apparent consumption of spirits in liters of ethanol *per capita* aged 15+ by state in groups over the years from 1950 to 2002.

**Figure 4.**
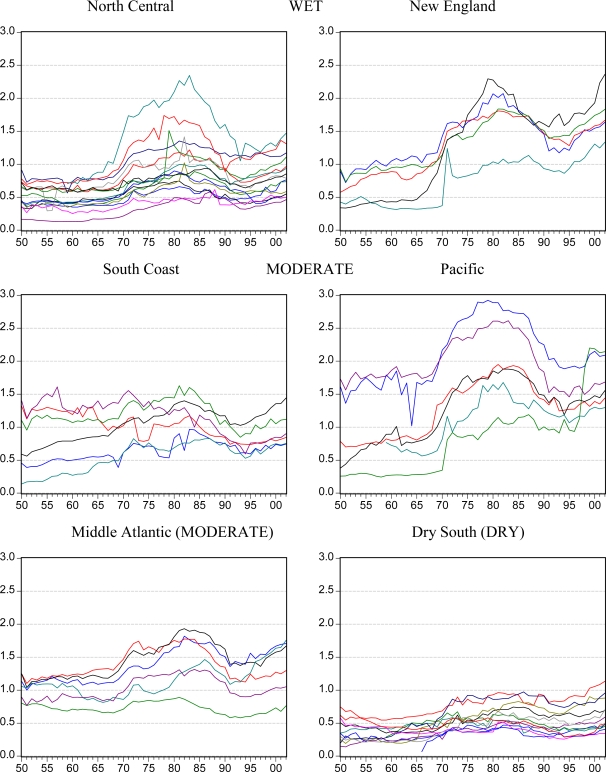
Apparent consumption of wine in liters of ethanol *per capita* aged 15+ by state in groups over the years from 1950 to 2002.

Figures 2, 3 and 4 illustrate trends in *per capita* beer, spirits and wine sales for each of the six state groupings. Although national trends lead to similarities between the groups and some persistent outlier states are seen, the six groupings are generally confirmed in terms of similarity in levels and trends. The Dry South states appear to be especially linked and different from the rest of the US.

In some cases, contrasting trends are evident such as the increasing beer consumption in the South Coast and Dry South states compared to the decreasing consumption in the Middle Atlantic states. For spirits, the shapes of most states trends are similar but the levels of consumption and the degree of the rising and falling pattern differ.

Figures summarizing the results of Granger Causality test comparisons of relationships between spirits and beer series within and outside of regional state groupings are presented in Table 2. The percentage of states within a grouping that have significant predictive relationships with each other is compared to the percentage of these relationships with states outside the grouping. These results do not consistently support the hypothesis that regional groupings will have more closely related beverage-specific trends in *per capita* apparent consumption. Some state groups were found to have a higher proportion of related state trends than outside relationships; spirits for Pacific and South Coast and beer for Dry South, North Central and New England. However, in all other cases the opposite was found or the proportion of relationships was the same. High percentages of relationships were found overall indicating that national trends are generally more important for US states than regional trends.

## 4. Discussion

Six groups of states were created by a qualitative inspection of several considerations: geographic proximity, recent (2005–7) survey measures of drinking patterns and *per capita* apparent consumption of alcohol in each jurisdiction. The degree to which these state drinking groups appear to be linked was assessed through time trends in *per capita* apparent consumption of beer, wine, spirits over the 1950 to 2002 period. Shared drinking measure trajectories are seen both within and across the defined groups suggesting some cohesiveness in regional drinking cultures as well as shared national trends. However, the results of Granger Causality tests designed to indicate predictive relationships between states did not find consistent evidence that state trends within these groups were more closely linked than trends between states in different groups for the beer and spirits beverage types. National trends in US alcohol consumption including rising consumption in the 1960’s and early 1970’s and declining consumption in the 1980’s as well as a shift from spirits to beer from about 1975 to 1995 can be seen in many of the states [13]. These changes, while not entirely understood, have been attributed to birth cohort differences in beverage preference and life-course drinking patterns [8,18] and to changes in alcohol policy such as the lowering and later raising of the minimum drinking age [19] and changes in policies and attitudes toward drunk driving in the 1980’s [20,21]. The national trends appear to dominate the state-grouping-specific trends. Despite the lack of clear validation for the grouping in terms of linked drinking patterns over time within the six groups, the groups are differentiated cross-sectionally and represent an empirically-based identification of US state regions varying in wetness *versus* dryness, suitable for use in the analyses of alcohol-consumption and related behaviors and outcomes. The simplified three group distinction between Wet, Moderate and Dry states represents a nested alternative grouping focused on drinking patterns and mean consumption levels.

Future studies at the individual-level should utilize and compare the efficacy of these two nested groupings to evaluate differences in drinking patterns, attitudes towards alcohol, and alcohol-related problems and outcomes in US surveys offering detailed measures of these constructs such as the National Alcohol Survey (NAS) and the National Epidemiologic Survey of Alcohol Related Conditions (NESARC). These groups should also be valuable for defining pooled models and matched control groups for cross-section time-series analyses of alcohol policy effectiveness and in estimating relationships between alcohol use measures and mortality or other outcomes in aggregate analyses at the US state level. Tests of the homogeneity of state time-series relationships between alcohol sales and specific mortality causes within and across these groups should also be undertaken to further evaluate group cohesiveness.

This paper updates the analyses of US drinking regions last considered over 20 years ago by Alcohol Research Group scientists [1,2]. The availability of state-specific alcohol pattern measures and long-term trend data on alcohol sales has greatly improved the information on which these regional groups are based. A large group of states in the North Central region and the New England states are found to clearly stand out in terms of having high proportions of heavy occasion drinkers and high *per capita* apparent consumption of alcohol, while a large group of mostly Southern states are found to stand out as relatively low on these measures. The groups incorporate a variety of differing alcohol availability policies and tax levels suggesting the importance of exploring cultural factors such as religion and religious involvement [7,22], race and ethnicity [23–25] and more detailed framings of ethnic origin [26]. These findings also suggest the importance of taking account of surrounding environments in alcohol abuse prevention efforts [27] and the implementation of effective alcohol policies [28], as appears to be particularly needed in the North Central and New England states.

## Figures and Tables

**Table 1.** States ranked by percentage of population 12+ who reported any 5+ in the past month in the 2005–6 NSDUH with additional age categories for past month 5+, percentage of drinkers and percentage of those with 5+ days among drinkers in the past month, *per capita* apparent alcohol consumption for 2005 and % reporting 5+/4+ in the past month in the 2007 BRFSS.

**State**	**%**					**Ethanol Gallons per capita age 15+ 2005**	**2007 BRFSS 5+/4+ Monthly age 18+ %**	**Group Name**
	**5+ Past Month age 12+**	**5+ Past Month ages 18–25**	**5+ Past Month age 26+**	**Drinker Past Month age 12+**	**Drinkers with 5+ in Past Month age 12+**			
North Dakota	30.32	56.49	26.99	58.24	52.06	2.74	23.2	NC
Wisconsin	29.41	53.60	27.42	63.14	46.58	2.96	23.4	NC
Montana	28.57	54.85	25.60	56.71	50.38	2.74	17.1	NC
Dist. of Columbia	28.32	50.58	26.10	60.25	47.00	3.89	16.1	none
South Dakota	28.14	51.78	25.88	58.70	47.94	2.48	17.3	NC
Minnesota	27.86	50.35	25.80	61.74	45.12	2.40	14.3	NC
Iowa	27.14	50.85	24.83	53.09	51.12	2.19	19.9	NC
Rhode Island	27.12	51.19	24.75	61.23	44.29	2.52	18.6	NE
Nebraska	26.69	49.97	24.17	54.03	49.40	2.35	18.0	NC
Vermont	25.99	53.20	22.99	60.42	43.02	2.62	17.9	NE
Illinois	25.46	46.27	23.81	52.47	48.52	2.33	19.5	NC
Kansas	25.43	47.82	22.81	53.19	47.81	1.94	14.6	NC
Michigan	25.37	47.92	23.64	56.2	45.14	2.17	18.5	NC
Wyoming	25.21	49.58	22.23	56.36	44.73	2.74	16.8	NC
Connecticut	25.14	49.83	23.08	60.77	41.37	2.32	17.8	MA
Massachusetts	25.01	52.44	22.13	57.85	43.23	2.55	17.6	NE
Colorado	24.68	47.24	22.56	58.59	42.12	2.69	17.3	NC
Ohio	24.45	47.30	22.40	51.57	47.41	2.00	17.1	NC
Texas	24.06	40.99	22.90	49.49	48.62	2.24	15.3	SC
Missouri	23.86	46.90	21.55	49.91	47.81	2.38	16.2	NC
New Hampshire	23.83	50.98	21.20	60.79	39.20	4.21	15.5	NE
Nevada	23.75	42.40	22.59	52.00	45.67	3.70	16.9	PA
Louisiana	23.53	36.79	22.80	49.07	47.95	2.66	13.4	SC
New York	23.47	43.77	21.53	54.75	42.87	1.99	15.2	MA
Pennsylvania	23.09	45.09	21.14	52.30	44.15	2.12	16.2	MA
Arizona	22.87	40.85	21.38	54.37	42.06	2.47	15.0	SC
**Total U.S.**	**22.82**	**42.02**	**21.20**	**51.37**	**44.42**	**2.27**	**15.8**	**US**
Washington	22.80	41.24	21.23	54.45	41.87	2.24	15.8	PA
Maine	22.43	45.82	20.28	54.76	40.96	2.47	15.9	NE
Florida	22.34	38.13	21.52	53.43	41.81	2.72	14.2	SC
New Jersey	22.29	45.17	20.24	56.47	39.47	2.32	13.6	MA
Idaho	21.89	37.51	20.48	46.52	47.06	2.53	14.7	PA
Kentucky	21.79	38.77	20.48	42.47	51.31	1.83	8.2	DS
Virginia	21.79	41.07	20.29	51.86	42.02	2.10	15.9	DS
Oregon	21.61	39.52	19.92	54.67	39.53	2.53	15.6	PA
Alaska	21.58	35.89	20.84	52.74	40.92	2.73	19.2	NC
Hawaii	21.41	40.37	20.06	46.70	45.85	2.56	18.6	PA
Delaware	21.29	42.59	19.14	51.51	41.33	3.30	18.6	MA
Oklahoma	21.14	37.08	19.60	41.85	50.51	1.51	12.5	DS
Indiana	21.10	41.05	19.19	49.40	42.71	2.00	15.6	DS
South Carolina	20.88	36.71	19.88	45.26	46.13	2.46	13.9	SC
California	20.81	37.75	19.41	50.33	41.35	2.29	16.9	PA
Arkansas	20.71	38.19	18.84	42.61	48.60	1.82	10.4	DS
Tennessee	20.52	39.91	18.95	42.41	48.38	1.89	9.2	DS
New Mexico	20.30	37.43	18.54	45.83	44.29	2.39	12.3	SC
Maryland	20.20	37.50	18.91	53.14	38.01	2.19	12.6	MA
Georgia	19.65	35.21	18.51	44.85	43.81	2.06	12.6	DS
North Carolina	19.46	35.79	18.24	43.58	44.65	1.97	12.3	DS
West Virginia	18.90	40.93	16.53	36.40	51.92	1.74	9.8	DS
Alabama	18.74	33.77	17.42	42.45	44.15	1.97	11.0	DS
Mississippi	18.40	31.84	17.33	36.93	49.82	2.25	11.3	DS
Utah	17.38	28.31	16.04	32.40	53.64	1.30	9.8	DS

Drinking Groups: PA- Pacific, NC- North Central, SC- South Coast, NE- New England, MA- Middle Atlantic, DS- Dry South.

**Table 2.** Proportion of Granger Causality Tests showing significant relationships between states within each state grouping compared to states outside the grouping for states in each group. Significant relationships indicate predictive value of one state’s spirits or beer series on another state’s series.

**Region**	**Number of states in group**	**Spirits within**	**Spirits outside**	**Beer within**	**Beer outside**
**Dry South** **Mid Atlantic** **North Central** **New England** **Pacific** **South Coast**	12 6 15 6 6 7	56.1% 66.7% 70.5% 60.0% **100.0%** **80.0%**	**73.1%** 68.1% 70.2% **72.2%** 74.4% 65.9%	**50.0%** 20.0% **53.3%** **80.0%** 40.0% 33.3%	44.0% **31.1%** 44.8% 50.4% **47.8%** **41.1%**
